# The Use of Hyaluronic Acid in the Treatment of Unilateral Vocal Fold Paralysis: A Systematic Review and Meta-Analysis

**DOI:** 10.7759/cureus.85728

**Published:** 2025-06-10

**Authors:** Khalid M Alkhalifah, Wejdan Ahmed, Hanan S Alnawmasi, Nouf Alshehri, Rola M Albalawi, Khaled A Aldawsari, Rahmah D Alatawi, Waleed Alhazmi

**Affiliations:** 1 Ear, Nose, Throat Department, Al-Rass General Hospital, Qassim Health Cluster, Al-Rass, SAU; 2 College of Medicine and Surgery, Tabuk University, Tabuk, SAU; 3 Department of Otolaryngology Head and Neck Surgery, College of Medicine, Qassim University, Buraydah, SAU

**Keywords:** glottal insufficiency, hyaluronic acid (ha), injection laryngoplasty, maximal phonation time (mpt), unilateral vocal fold paralysis (uvfp)

## Abstract

Unilateral vocal fold paralysis (UVFP) is a neurological condition characterized by glottal insufficiency, commonly resulting from iatrogenic damage sustained during head and neck surgery. Hyaluronic acid (HA), a naturally occurring glycosaminoglycan, has been shown to influence vocal cord function positively. This study aimed to evaluate the effectiveness of HA in treating UVFP. A systematic review and meta-analysis were conducted following the PRISMA guidelines (PROSPERO ID: CRD42024560084). Literature searches were performed in PubMed, Cochrane Library, Web of Science, and Google Scholar up to August 2024 using a comprehensive combination of terms related to HA and UVFP. Two independent reviewers conducted study selection, data extraction, and quality appraisal using the Newcastle-Ottawa Scale for observational studies and the Cochrane Risk of Bias tool for randomized controlled trials. Thirty-two studies involving 1,917 adult patients were included. Outcomes assessed included quality of life (QOL), maximal phonation time (MPT), and normalized glottal gap area (NGGA). Meta-analysis demonstrated significant short- and medium-term improvements in patient-reported QOL following HA injection (standardized mean difference [SMD]=0.87, 95% CI [0.65-1.09]; p < 0.0001; I²=0%). Medium-term MPT also improved significantly (SMD=0.45, 95% CI [0.16-0.74]; p=0.003; I²=2%). Similarly, NGGA improved in the medium term (SMD=0.51, 95% CI [0.19-0.83]; p=0.002; I²=0%). Adverse events were rare and mostly minor, including transient dysphonia and inflammation. The overall risk of bias was low in most included studies. HA injection laryngoplasty appears effective in improving vocal function and quality of life in patients with UVFP in the short to medium term. However, the current evidence is limited by variable follow-up durations, technique heterogeneity, and insufficient long-term data. Future studies should explore patient-specific outcomes, standardized intervention protocols, long-term efficacy, and safety across diverse populations.

## Introduction and background

Unilateral vocal fold paralysis (UVFP) is a common neurological condition characterized by glottal insufficiency, often resulting from iatrogenic injury to the recurrent laryngeal nerve (RLN) during thoracic, thyroid, or cervical spine surgery [1]. Patients with UVFP may often experience hoarseness, dysphonia, and aspiration, which can significantly impair quality of life. While some cases demonstrate spontaneous recovery, persistent paralysis may necessitate surgical intervention [1]. Injection laryngoplasty (IL) and open laryngeal framework surgery are two surgical methods that have shown effectiveness in swiftly enhancing vocal function by relocating the paralyzed vocal fold closer to the midline of the glottis [1,2].

As methods and alternatives for therapy continue to evolve, there is a need for ongoing research to determine the most effective treatment options for various scenarios [3,4]. Multiple injectable materials have been employed in IL, including collagen, autologous fat, calcium hydroxylapatite, and Teflon. However, hyaluronic acid (HA) has gained prominence due to its biocompatibility, favorable viscoelastic properties, and low immunogenicity [5,6]. Its biomechanical resemblance to the native vocal fold extracellular matrix may offer advantages in restoring vibratory function compared to other agents [5]. Despite its widespread use, the comparative efficacy and safety of HA remain incompletely defined, particularly across diverse patient populations and follow-up durations.

Vocal folds treated with HA-based chemical components exhibit viscoelastic characteristics similar to those of healthy vocal folds, despite variations in the types and molecular sizes of HA fillers [7]. This favorable safety profile has contributed to the widespread use of HA. However, injection procedures, outcomes, and side effects vary across different studies [1-7]. Various formulations of hyaluronic acid, such as Restylane, Juvederm, and Silk-HA, have been used in injection laryngoplasty, but the existing literature does not clearly differentiate outcomes by HA type. As a result, this review focuses on pooled outcomes across all HA variants. The diversity of HA products may introduce variability in efficacy or duration of benefit, which remains an important but understudied factor. Therefore, this systematic review aims to compile information on the use of HA-based components for the treatment of UVFP. Additionally, a meta-analysis is conducted to evaluate clinical outcomes and examine the effects of HA on UVFP treatment. The findings of this study are expected to assist healthcare professionals in enhancing their clinical approaches to patient care.

## Review

Materials and methods

Literature Search

This systematic review was registered with the Prospective Register of Systematic Reviews (PROSPERO) (ID: CRD42024560084) [8]. The review was conducted in accordance with PRISMA 2020 [9]. There were no restrictions on publication year; studies from all years up to August 2024 were included. The search was not updated after August 2024, and this is noted as a limitation. The search strategy was pilot-tested for retrieval effectiveness but not formally validated. A literature review was performed across three principal medical health databases and an additional source. The four databases searched include PubMed, the Cochrane Library, Web of Science, and Google Scholar. The search terms were (hyaluronic acid OR HA OR hyaluronate) AND (unilateral vocal fold palsy OR unilateral vocal cord palsy OR unilateral vocal fold paralysis OR unilateral vocal cord paralysis OR unilateral vocal fold immobility OR unilateral vocal cord immobility OR recurrent laryngeal nerve paralysis) used in combination.

Eligibility Criteria

The inclusion criteria for this study are as follows: Adult patients (≥18 years) with UVFP treated using HA injection laryngoplasty reported outcomes related to voice quality, glottal closure, phonation time, or adverse events and were published in English in peer-reviewed journals.

The exclusion criteria include studies involving pediatric patients or those under 18 years old, animal studies, case reports, conference abstracts, gray literature, and non-English publications and unpublished data, which were excluded due to a lack of peer review and outcome verification.

Selection of Articles and Data Extraction

Two proficient reviewers independently evaluated the titles and abstracts retrieved using the search technique utilizing Rayyan software [44]. To ascertain ultimate eligibility, each author independently examined the complete contents of the chosen abstracts. In instances of discord, a third reviewer was engaged to promote dialogue and achieve consensus. The data obtained from each study encompasses: the first author's name, article title, publication year, journal, study design, country, patient count, age (mean and standard deviation or range), material designation, anesthesia type, injection technique, injection guidance, cutaneous injection route, vocal fold injection, injected volume (cc), follow-up duration (months), evaluation methodologies, and adverse events [10].

Statistical Data Analysis

The statistical analysis was performed utilizing Review Manager (RevMan Version 5.4.2). To address data heterogeneity and mitigate the impact of missing information, a random-effects model was used, employing the last observation carried forward method. Heterogeneity was assessed using the I², Tau², and Chi² test statistic, with significant heterogeneity defined as an I² value exceeding 50%. The methodological quality and risk of bias in the studies included were evaluated using appropriate tools. Due to variation in the reporting of the change in maximal phonation time (MPT) and normalized glottal gap area (NGGA), with most of the studies reporting the overall effect size, we standardized data by converting reported data into odds ratio using logistic regression. The Newcastle-Ottawa Scale (NOS) was applied to assess prospective and retrospective cohort studies, cross-sectional studies, and case-control studies' risk of bias; the total scoring was put into three categories (0-3-high risk of bias, 4-6- moderate risk of bias, 7-9-low risk of bias). For randomized controlled trials, the Cochrane Risk of Bias Assessment tool was utilized. Similarly, because more than 10 studies were included in the meta-analysis, a funnel plot was used to analyze the possibility of publication bias using Egger's test statistics.

Results

**Figure 1:**
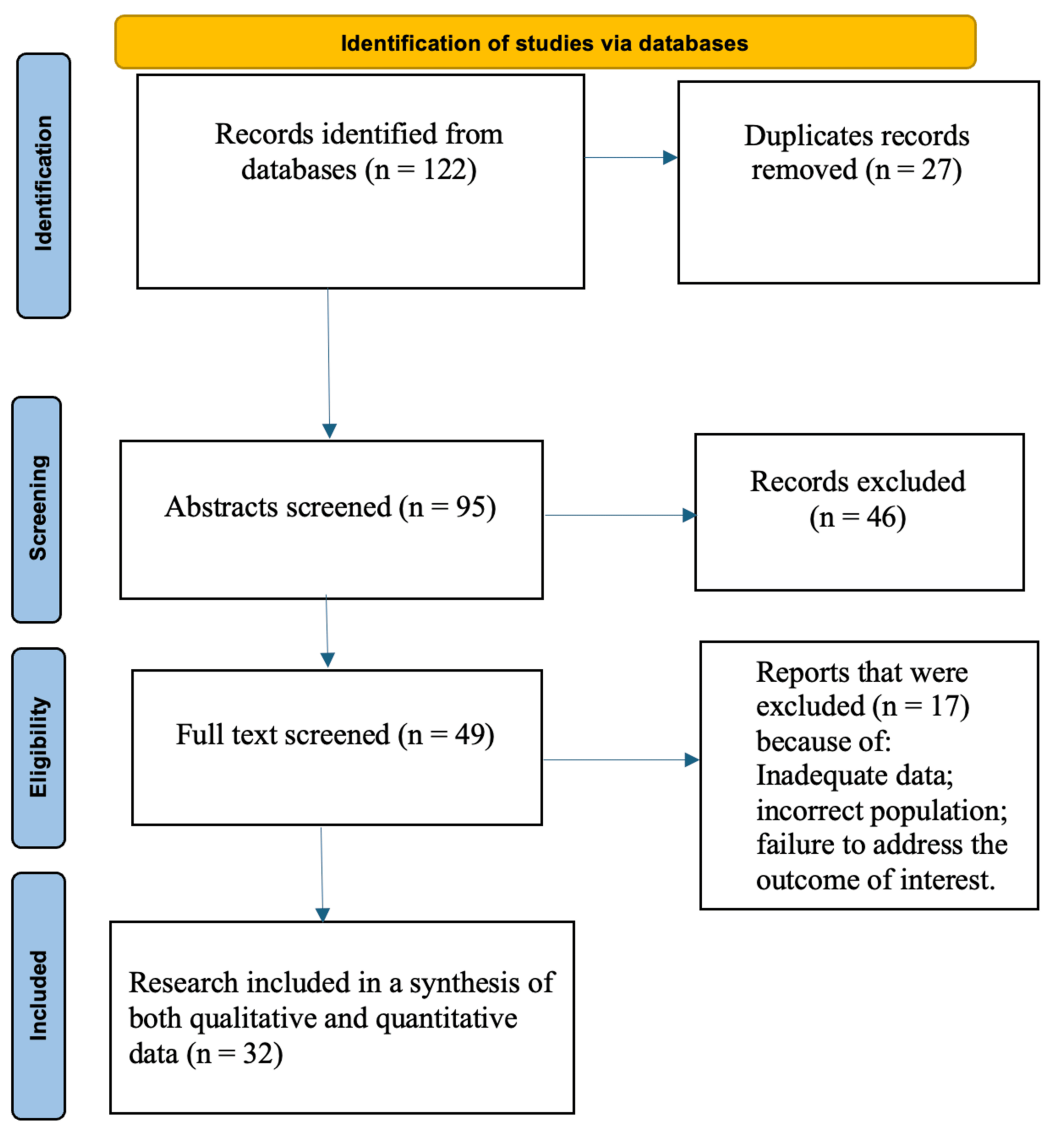
PRISMA flow diagram.

Out of the 122 papers obtained from the online database search, 27 duplicate studies were identified and removed. After screening the study titles and abstracts, 46 papers were excluded. The remaining 49 publications were evaluated in full text for eligibility. At this stage, 17 investigations were excluded due to inadequate data, incorrect population, or failure to address the outcome of interest. Ultimately, 32 studies were included in the qualitative and quantitative synthesis (Figure 1).

Qualitative Synthesis

The features of the studies that were part of this systematic review are shown in Table 1. The included studies were all published in English and were carried out in different parts of the world; eight studies were conducted in Taiwan, seven in the USA, four in Turkey, three in Hong Kong, and two each in Poland and Korea, while Singapore, Germany, Canada, Malaysia, Egypt, and Sweden had one study each, respectively. The included studies comprised a total sample of 1917 participants; the study with the lowest sample size had five participants, while the study with the highest number of participants had 141 participants. Different forms of hyaluronic acid were used as the intervention in all of the included studies.

**Table 1:** Characteristics of the included studies. UVFP: Unilateral paralysis of the vocal folds Laryngoplasty with hyaluronic acid injection, or HIL HL: high level, HR: high-rising Clinical skill laboratory, or CSL, antibodies against phospholipids (APA), lactated Ringer's solution (LR), hyaluronic acid/dextranomer, or HA/D otorhinolaryngology (ORL), visual field index (VFI), and vocal handicap index (VHI) Hyaluronic acid, or HA, interleukins, or IL Vocal fold injections guided by laryngeal electromyography, or LEVFI Voice Range Profile (VRP) Quality of life, or QOL Thyroarytenoid-LCA-lateral cricoarytenoid muscle complex, UVCP, TA, and laryngeal electromyography, or LEMG, CM: conservative medicine Normalized glottal gap area (NGGA) PL: discomfort following surgery, maximum phonation time, or MPT; phonation quotient, or PQ Mean air flow rate, or MAFR GRBAS: strain, asthenia, breathiness, roughness, and grade Licensed Professional in the Healing Arts, or LPHA

Authors	Country	Study Design	Sample size	Intervention	Inclusion	Results/Outcome
HertegÃ¥rd et al., 2002 [11]	Sweden	Prospective, randomized controlled study	83	Hylan B gel (60) + Collagen (24)	Individuals with glottal insufficiency received injections of bovine collagen and hylan B gel. After six, twelve, and one year of treatment, the patients' status was assessed.	Both the collagen and hylan B gel groups showed improvements in patient self-ratings and glottal closure 12 months following injections. Hylan B gel users retained their glottal region vibration amplitude and fluctuations, had a longer maximum phonation time, and experienced reduced resorption near the margin of the injected vocal fold.
Lau et al., 2010 [12]	Singapore	Prospective, randomized controlled study	17	Restylane (8) + Perlane (9)	Individuals who needed medialization for UVCP and were over the age of eighteen were eligible to participate.	Transcartilage IL with larger particle sizes is safe, durable, and has a six-month shelf life. For short-term mediation, LPHA may be considered for UVCP patients seeking a medium-term intervention.
Friedman et al., 2010 [13]	USA	Retrospective cohort study	35	Hyaluronic acid	Patients who had been diagnosed with unilateral vocal cord paralysis and had dysphonia within a year of the onset of paralysis were given a paraglottic injection.	In 32 patients (62.5%), early injection medialization prevented open-neck phonosurgical repair; follow-up varied from 4.0 to 41.8 months; three patients who underwent late injection did not avoid reconstruction.
Redolf et al., 2012 [14]	Germany	Prospective cohort study	19	Hyaluronic acid	UVP patients received assessments prior to surgery, six weeks after the procedure, and six months after vocal fold augmentation with Restylane. The patients used the Voice Handicap Index to rate themselves.	After a year, 58% of the patients still had acceptable voice qualitye, but after six weeks, 42% of them had serious voice impairment that needed further treatment, such as thyroplasty or laryngectomy.
Wang et al., 2012 [15]	Taiwan	Prospective cohort study	20	Hyaluronic acid	Paralyzed thyroarytenoid muscle patient received an injection of 1.0cc of HA before to LEMG. A 3-month follow-up was performed by twenty patients, and a 6-month follow-up by sixteen patients.	Significant decreases in NGGA, MPT, VHI, and other evaluated data, such as PQ, MAFR, GRBAS scale, and choking severity, were observed after all patients successfully underwent the surgery without any issues.
Szkie Å‚ kowska et al., 2013 [16]	Poland	Non-Randomized non-comparative study	25	Hyaluronic acid	Twenty-five dysphonia patients had evaluations of their laryngology, phoniatric, and acoustic voice performed prior to and following surgery, as well as one, three, and six months later.	In every case, glottic closure was attained following surgery. After three months, vocal fold vibration returned in eight more cases, rising to fifteen. A month following surgery, notable changes in perceived voice quality were noted. The results of multidimensional voice analysis indicated that frequency characteristics improved quickly.
Oguz et al., 2013 [17]	Turkey	Retrospective cohort study	17	Hyaluronic acid	HA-D injections were given to 17 UVFP patients, 4 men and 13 women, ages 34 to 56. The long-term outcomes were assessed using acoustic analysis and the GRBAS scale, both subjectively and objectively.	Fundamental frequency, jitter, and the noise-to-harmonics ratio all dropped postoperatively, but shimmer was steady. Nevertheless, no appreciable variations were seen in the acoustic analysis findings or GRBAS scale parameters.
Upton et al., 2013 [18]	USA	Prospective cohort study	30	Juvederm ultra plus gel	patients who underwent Juvederm Ultra Plus gel injection laryngoplasty over a 20-month period	Twenty-seven patients had demonstrated significant improvements at one and four months on all outcome measures; one patient, however, needed intravenous steroid therapy for delayed glottic inflammation.
Halderman et al., 2014 [19]	USA	Retrospective cohort study	64	SPHA (Restylane)	Patients who received Restylane injection medialization in an office setting during a two-year period	An analysis of 64 individuals' 82 Restylane injections showed five adverse events, an average benefit of 12.2 weeks, and a tendency away from permanent medialization.
Fang et al., 2014 [20]	Taiwan	Prospective cohort study	20	Restylane	Patients diagnosed with UVFP within the past six months underwent laryngeal electromyography, voice laboratory analysis, a voice outcome assessment, and initial and follow-up videolaryngostroboscopy.	The only indicator of post-operative pain (PL) in patients undergoing conservative treatment was normalized glottal gap area (NGGA). Those whoâ€™s first NGGA was more than 7.36 had a higher prevalence of depression and poorer social and emotional functioning than those who received an early hyaluronic acid injection.
Pei et al., 2015 [21]	Taiwan	Randomized controlled study	14	Restylane	At baseline and six months following a single hyaluronate injection (HI) or conservative therapy (CM), the quality of life, voice outcomes, and health-related quality of life of 29 UVFP patients were evaluated.	At the conclusion of the follow-up, the HI group outperformed the CM group in the majority of quality-of-life areas with respect to improvements in mental health quality of life.
Wang et al., 2015 [22]	Taiwan	Prospective cohort study	60	Perlane	Individuals who got UVFP hyaluronic acid VF injection under LEMG guidance at a tertiary referral medical hospital between March 2010 and February 2013.	After one month, six months, and the most recent follow-up assessment, notable changes in the characteristics of glottal closure were noted. A mean normalized glottal gap area, a reduction in the phonation quotient, an extension of the maximal phonation time, and a decrease in the airflow rate were all observed. There was also a noticeable improvement in the Voice Handicap Index score.
Fang et al., 2015 [23]	Taiwan	Retrospective cohort study	34	Restylane	After thoracic surgery, patients with UVFP who got office-based HA injections underwent a battery of tests at baseline and one month after the injection.	After thoracic surgery-related UVFP, in-office HA intracordal injection significantly improved voice quality, voice laboratory analysis, and generic quality of life areas without any complication.
Wong et al., 2016 [24]	Hong Kong	Non-Randomized non-comparative study	11	Restylane, Galderma, Switzerland	Eleven UVFP patients who had videostroboscopic tests, vowel sustain, and texts read aloud before, during, and after laryngoplasty.	For UVFP patients, injectable laryngoplasty results in immediate and durable improvements in voice quality and quality of life that endure for up to three months following surgery, even if there is no significant change in fundamental frequency.
Kara et al., 2018 [25]	Turkey	Retrospective cohort study	5	Hyaluronic acid	Patients with dysphonia who experienced ineffective coughing, aspiration, and UVCP	Five UVCP patients experienced poor cough reflex and oral aspiration. Patients had lung excision surgery without any problems or fatalities. ILP may be an active treatment for UVCP before resection.
Kim et al., 2018 [26]	Korea	Prospective cohort study	50	Hyaluronic acid	Patients with VFP were evaluated for vocal fold function using acoustic analysis, auditory-perceptual testing, visual judgment	Following IL therapy with hyaluronic acid, patients with VFP showed notable improvements in self-questionnaire, auditory, visual, and acoustic assessments, in addition to good vocal fold function improvement.
Ng et al., 2018 [27]	Hong Kong	Non-Randomized non-comparative study	30	Restylane	Investigated the patients' voices both before and after surgery in terms of their appearance, perception, sound, and aerodynamics.	In addition to enhancing QOL, tone production, pitch characteristics, and perceptual voice quality, IL effectively treats UVFP. Future study ought to focus on enhancing speech comprehension and general communication skills.
Pei et al., 2018 [28]	Taiwan	Prospective cohort study	85	Hyaluronate (Restylane)	UVFP patients, including 68 who had intracordal hyaluronate injections, assessed VRP, acoustic and aerodynamic assessments, and TA-LCA peak turn frequency.	Patients with UVFP showed increased voice pitch prediction (VRP) performance following intra-cordal hyaluronate injection, suggesting that VRP can predict impairment of neuromuscular function and glottal gap. An additional indication of the validity of VRP is a strong response to voice improvements after laryngoplasty.
Bertroche et al., 2019 [29]	USA	Retrospective cohort study	59	Juvederm (hyaluronic acid)	Patients who underwent Juvederm laryngoplasty in assessing their longevity and effectiveness in Voice-Related Quality of Life index scores,	Juvederm injection laryngoplasty is useful for treating vocal cord atrophy, paresis, and paralysis. Furthermore, it improves Voice Related Quality of Life scores with an average duration of 10.6 months.
Liu et al., 2020 [30]	Canada	Retrospective cohort study	121	Hyaluronic acid (HA)	Individuals who, from August 2017 to December 2018, had their first LEVFI.	Individuals with voice impairment who receive in-office LEVFI, a novel approach for treating glottic insufficiency, had considerably better voice outcomes with a high completion rate and no major problems.
Enver et al., 2021 [31]	Turkey	Retrospective cohort study	476	Hyaluronic acid (HA) and Hyaluronic acid with Dextranomer (HA-D)	Patients who, between January 2005 and September 2016, underwent vocal fold augmentation with HA injections at nine different hospitals.	Nine complication, predominantly inflammatory reactions with dysphonia and dyspnea, were found among theÂ patients receiving hyaluronic acid under general anesthesia. Three patients were admitted to the hospital and treated with antibiotics and systemic steroids.
Jeong et al., 2020 [32]	Korea	Retrospective cohort study	40	Hyaluronic acid (HA)	Patients with UVFP who received IL as their main treatment were assessed.	After IL, voice parameters were similar in all groups; however, voice treatment improved them for a six-month period. Voice therapy may be helpful for UVFP patients, as seen by the lower overall voice handicap index-30 scores among these patients.
Chow et al., 2021 [33]	Malaysia	Non-Randomized non-comparative study	29	Hyaluronic acid	Individuals diagnosed with UVFP who underwent injectable laryngoplasty surgery within a half-year after the onset of symptoms	Early transthyrohyoid injection laryngoplasty significantly improved voice and quality of life without causing any major complications in patients with UVFP. It was a safe and effective procedure.
Chang et al., 2021 [34]	Taiwan	Retrospective case series study	141	Hyaluronic acid	Patients who have UVFP related to surgery	Patients undergoing lung surgery had greater jitter, reduced involvement of the external laryngeal nerve, and left-sided UVFP. Following office-based hyaluronate injection, results improved, with the group undergoing lung surgery seeing the biggest improvement. It is advised to seek early assistance in order to enhance voice function and overall well-being.
Miaskiewicz et al., 2022 [35]	Poland	Retrospective cohort study	75	Hyaluronic acid	Individuals receiving an injection of either HA or CaHA for dysphonia brought on by unilateral paralysis of the vocal folds	Comparably, the glottal gap improved in both groups, with the CaHA group's mean change in VHI being 29.14 and the HA group's mean change being 22.88. As for the amount of reaugmentations following injection laryngoplasty with CaHA as opposed to HA, there were no long-term variations in voice results.
Dwyer et al., 2022 [36]	USA	Retrospective cohort study	58	Hyaluronic acid	Patients receiving Silk-HA injections from July 2020 to December 2021 as a treatment for unilateral paralysis of the vocal folds.	After three months, Silk-HA shows promise for continued improvement in glottic insufficiency, with an improvement in voice outcomes overall. Longevity is still unknown, though, and patients should be warned about the possibility of dyspnea and airway edema.
Gao et al., 2024 [37]	USA	Prospective cohort study	17	Hyaluronic acid	UVFP patients who chose to receive treatment with VFIM and silk-HA	Twelve months following their silk-HA injection, seven patients continued to benefit from the treatment, with a median improvement of 19 in the VHI-10. Glottal closure, either complete or touch, was observed in five cases, followed by regression. Sustained therapeutic response necessitates more investigation.
Dwyer et al., 2024 [38]	USA	Retrospective cohort study	134	Silk hyaluronic acid	Voice samples from patients with UVFP taken before and after intervention were used to track changes in VHI-10 over time.	An injectable that is both safe and efficient for treating UVFP and augmenting VFI is called silk-HA. Re-medialization procedures are advised in light of the lower ratings observed in postoperative follow-ups. For several months, and even after a year, patients should continue to improve.
Alaskarov et al., 2024 [39]	Turkey	Non-Randomized non-comparative study	40	Hyaluronic acid/dextranomer	The study comprised patients undergoing HA/D injections for UVFP.	In patients with UVFP, HA/D injection laryngoplasty was shown to be helpful due to the significant improvement in voice and swallowing functions (excluding maximal phonation length) and the lack of significant change between the preoperative value and the postoperative 24-month period.
Sheikhany et al., 2022 [40]	Egypt	Prospective cohort study	19	Hyaluronic acid (Perfecta deep)	Patients who arrived at the ORL outpatient clinic with unilateral vocal fold paralysis	Following surgery, CSL, APA, and phonatory gap significantly improved in both groups, with group B demonstrating superior outcomes six months later. Both treatments improved voice and glottal closure, with resorption noted, and no significant side effects were reported.
Au et al., 2013 [41]	Hong kong	Prospective cohort study	14	Restylane	Individuals with UVFP who have laryngoplasty injections with hyaluronic acid (Restylane)	Cantonese-speaking IL injection significantly enhanced the signal-to-noise ratio, reduced shimmer and jitter, and enhanced the production of HL, HR, and LR tones in UVFP patients. The enhancement continued for at least three months.
Pei et al., 2019 [42]	Taiwan	Prospective case series study	75	Restylane	Individuals with UVFP who received a single HIL within six months of their initial outpatient appointment.	Individuals with UVFP who had substantial anomalies in these domains both before and after surgery saw considerable improvements in glottal conformation, voice, and aerodynamics following hyaluronate injection laryngoplasty (HIL). Speech treatment is necessary since people with UVFP continued to have inferior aerodynamics even after HIL.

Quantitative synthesis (meta-analysis)

Assessment of the Patients Quality of Life

**Figure 2:**
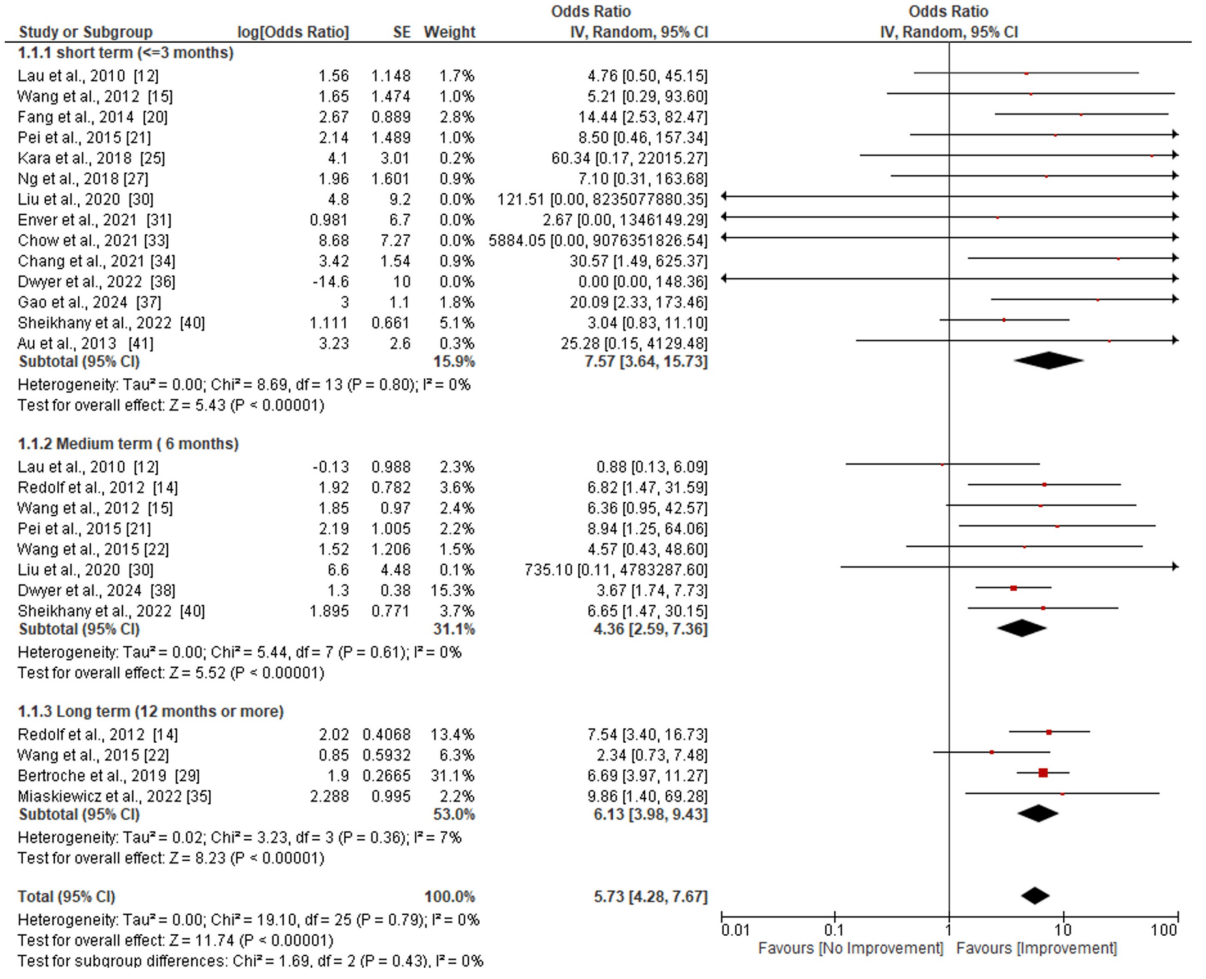
Subgroup analysis of overall quality of life following hyaluronic acid injection laryngoplasty.

Figure 2 shows the patients' overall quality of life following hyaluronic acid injection therapy. Evaluation was done periodically on the basis of the follow-up period, which was categorized into short- (<=3 months), medium-(<=6 months), and long-term (12 months or more). Fourteen studies [12,15,20,21,25,27,30,31,33,34,36,37,40,41] were included in the assessment of patients’ quality of life in the short term (<=3 months). The analysis shows that the use of hyaluronic acid injection in the short term significantly led to the improvement of patients’ quality of life (p<0.00001). Similarly, the heterogeneity of the included studies was very low (Tau^2^=0.00, Chi^2^=8.69, I^2^=0%, p=0.80). Assessment of the medium term included eight studies [12,14,15,21,22,30,38,40]. The results of the medium term also reveal a significant improvement in quality of life (p<0.00001) after injection. The heterogeneity of included studies was also found to be low (Tau^2^=0.00, Chi^2^=5.44, I^2^=0%, p=0.61). Nonetheless, only four studies [14,22,29,35] reported long-term effects. The results also indicated an improvement in quality of life after injection within 12 months of follow-up (p<0.00001). The assessed studies also revealed a low level of heterogeneity (Tau^2^=0.02, Chi^2^=19.10, I^2^=0%, p=0.79).

Assessment of the Voice Recording

**Figure 3:**
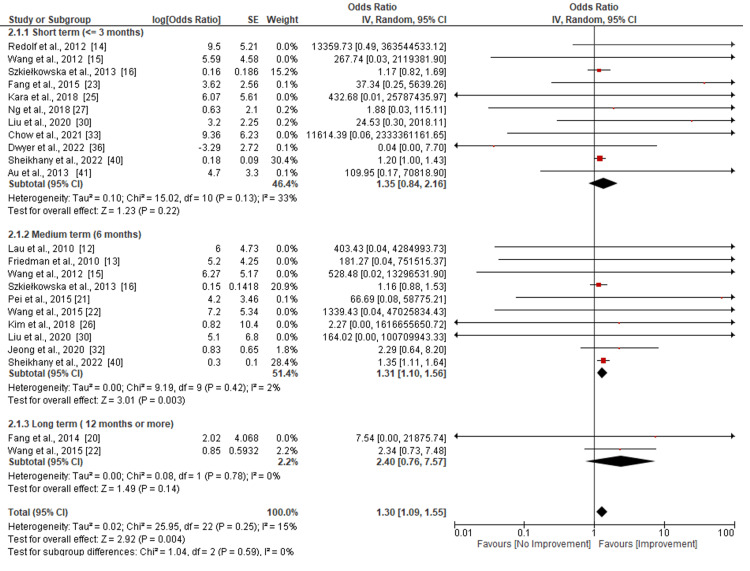
Subgroup analysis of the total maximal phonation time (MPT) following hyaluronic acid injection laryngoplasty.

Figure 3 shows the evaluation of voice recording following hyaluronic acid injection therapy intervention. The evaluation was done based on the follow-up period adopted by most of the researchers, which was in most cases categorized into short- (<=3 months), medium- (<=6 months), and long-term (12 months or more). Eleven studies [14-16,23,25,27,30,33,36,40,41] were included in the assessment of the effect of injection on MPT in the short term (<=3 months). The analysis shows that the use of hyaluronic acid injection in the short term did not have a significant increase in MPT (p=0.22), while the heterogeneity of the included studies was moderately low (Tau^2^=0.10, Chi^2^= 10.02, I^2^=33%, p=0.13). Further, the assessment of the medium term included ten studies [12,13,15,16,21,22,26,30,32,40]. The results of the medium-term reveal a significant increase in MPT (p<0.003) after injection. The heterogeneity of included studies was also found to be low (Tau^2^=0.00, Chi^2^=9.19, I^2^=2%, p=0.42). Nonetheless, only two studies [20,22] reported long-term effects. These results indicated that there was no significant increase in MPT after injection in the long term (p=0.14). Further, a low level of heterogeneity (Tau^2^=0.00, Chi^2^=0.08, I^2^=0%, p=0.78) was revealed by the included studies.

Assessment of the Image or Video Analysis 

**Figure 4:**
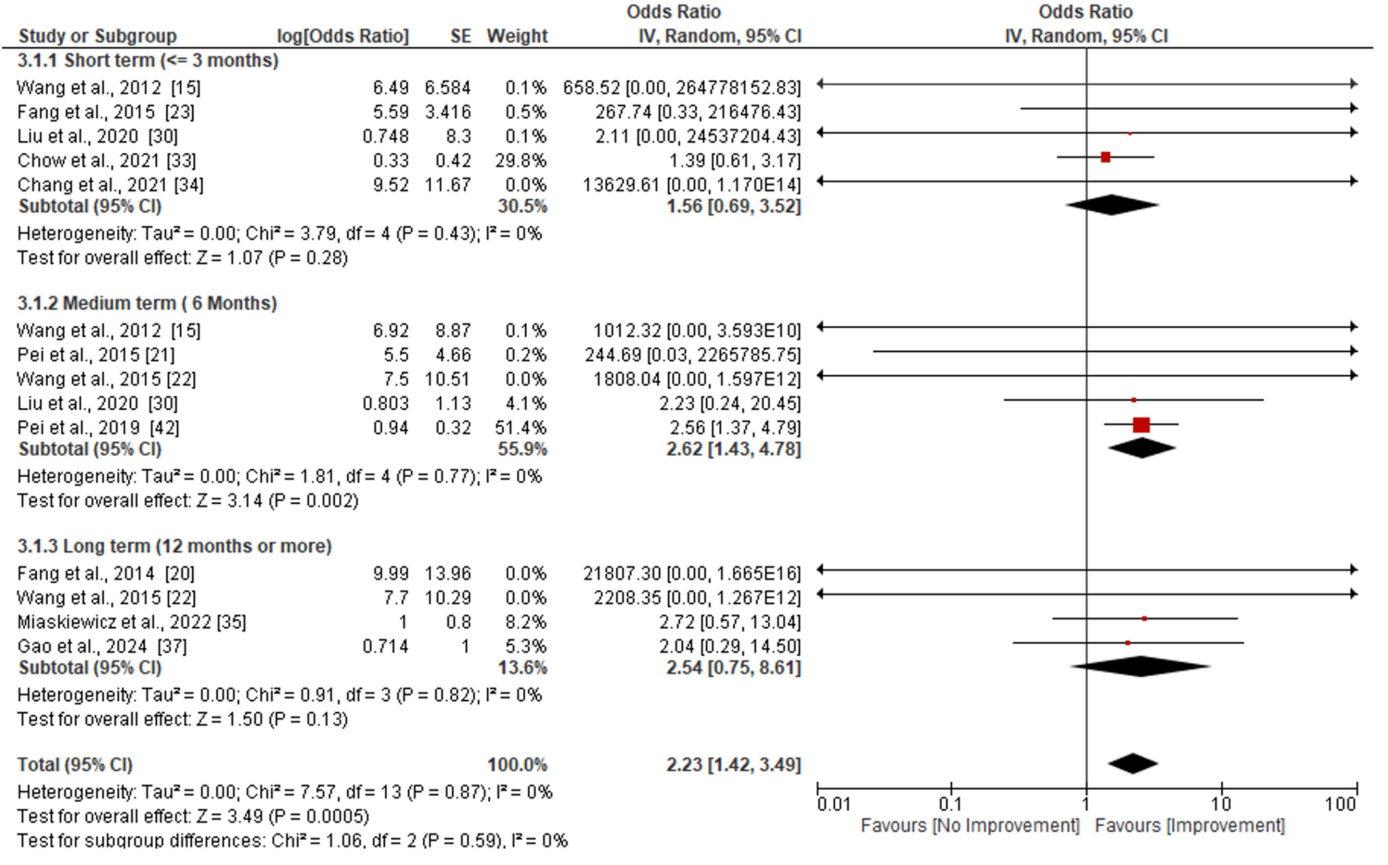
Subgroup analysis on the total normalized glottal gap area (NGGA) following hyaluronic acid injection laryngoplasty.

Figure 4 shows the assessment of the image and video analysis following the hyaluronic acid injection therapy intervention. The assessment was done based on follow-up intervals employed by the researchers; this was categorized into short- (<=3 months), medium-(<=6 months), and long-term (12 months or more). Five studies [15,23,30,33,34] were included in the assessment of total NGGA effect sizes in the short term (<=3 months). The analysis shows that the use of hyaluronic acid injection in the short term did not yield a significant improvement in NGGA (p=0.28). Similarly, the heterogeneity of the included studies was very low (Tau²=0.00, Chi²=3.79, I²=0%, 0.43). Assessment of the medium term also included five studies [15,21,22,30,42]. Contrary to short-term results, the results of the medium-term revealed a significant improvement in total NGGA (p=0.002) after injection. The heterogeneity of included studies was also found to be low (Tau^2^=0.00, Chi^2^=1.81, I^2^=0%, p=0.77). In the assessment of long-term effects, four studies that reported the results were included [20,22,35,37]. Similarly, in the short term, this result also indicated that there was no significant improvement in the total NGGA after injection within 12 months of follow-up (p=0.13). The assessed studies also revealed a low level of heterogeneity (Tau²=0.00, Chi²=0.91, I²=I2=0%, p=0.82).

Risk of bias assessment

The quality of cohort, prospective, and non-randomized observational studies was evaluated using the Newcastle-Ottawa Scale (NOS) (Farrah et al. [10]). As presented in Table 2, the NOS was used to assess studies based on three domains: selection, comparability, and outcome. Each study was rated to a maximum of 9 scores. Out of the 32 included studies, 24 studies were found to have a low risk of bias based on their strong performance across all NOS domains [15-25,29-30,31-39]. These studies demonstrated adequate representativeness, appropriate exposure ascertainment, sufficient follow-up, and well-addressed comparability. The remaining eight studies assessed were found to have moderate risk of bias [13,14,26,28,40-42], primarily due to limitations in selection criteria or comparability of cohorts as well as due to unclear or poor reporting of exposure determination, inadequate cohort selection, or insufficient follow-up. The fact that most of the studies had a low risk of bias, with only eight out of thirty-two studies having a moderate risk and none of the studies assessed having a high risk of bias, implies that the included studies were of good quality. Therefore, the results of the study can be reported with confidence.

**Table 2:** Risk of bias based on quality assessment of cohort retrospective and prospective studies-NOS. Q1. Is the exposure cohort representative? (1) Q2. How was the non-exposure cohort chosen? (1) Q3. Determination of the exposure? (1) Q4. Proof that the desired outcome did not exist at the outset of the research? (1) Comparability Q5. Is the cohort comparable based on the analysis or design? (2) Outcome: Q6: Evaluation of the result? (1) Q7: Was the Follow-Up Period Sufficient for Results to Arise? (1) Q8: Sufficient Follow-Up for Cohorts? (1) For every item in the selection and outcome categories, a study received up to one score (1). However, comparability received a rating of up to two scores (2). For selection and outcome, (1) shows a low risk of bias in each item. On the other hand, for comparability, (2) shows a low risk of bias, while (0) shows a high risk of bias and (-) an unclear risk of bias.

Authors	Selection				Comparability	Outcome			Total score (0-3-high risk, 4-6-moderate risk, 7-9-low risk)
	The exposed cohort's representativeness	The non-exposed cohort's selection	Determination of exposure	Evidence that the desired outcome Was not present at the outset of the research	Comparability of cohorts based on the analysis or design	Evaluation of results	Was the follow-up period sufficient for results to arise?	Sufficient follow-up for cohorts	
Friedman et al., 2010 [13]	1	0	0	-	1	0	1	1	4/9
Redolf et al., 2012 [14]	1	0	0	-	1	0	1	1	4/7
Wang et al., 2012 [15]	1	1	1	-	2	1	1	1	8/9
Szkiełkowska et al., 2013 [16]	1	1	1	-	2	1	1	1	8/9
Oguz et al., 2013 [17]	1	1	1	-	1	1	1	1	7/9
Upton et al., 2013 [18]	1	1	1	-	2	1	1	1	8/9
Halderman et al., 2014 [19]	1	1	1	-	2	1	1	1	8/9
Fang et al., 2014 [20]	1	1	1	-	2	1	1	1	8/9
Pei et al., 2015 [21]	1	1	1	-	1	1	1	1	7/9
Wang et al., 2015 [22]	1	1	1	-	2	1	1	1	8/9
Fang et al., 2015 [23]	1	1	1	-	2	1	1	1	8/9
Wong et al., 2016 [24]	1	1	1	-	1	1	1	1	7/9
Kara et al., 2018 [25]	1	1	1	-	2	1	1	1	8/9
Kim et al., 2018 [26]	1	1	1	-	2	1	1	1	8/9
Ng et al., 2018 [27]	1	0	0	-	2	1	1	1	6/9
Pei et al., 2018 [28]	1	0	0	-	2	1	1	1	6/9
Bertroche et al., 2019 [29]	1	0	0	-	1	1	1	1	5/9
Liu et al., 2020 [30]	1	1	1	-	2	1	1	1	8/9
Enver et al., 2021 [31]	1	1	1	-	2	1	1	1	8/9
Jeong et al., 2020 [32]	1	1	1	-	2	1	1	1	8/9
Chow et al., 2021 [33]	1	1	1	-	1	1	1	1	7/9
Chang et al., 2021 [34]	1	1	1	-	2	1	1	1	8/9
Miaskiewicz et al., 2022 [35]	1	1	1	-	1	1	1	1	7/9
Dwyer et al., 2022 [36]	1	1	1	-	2	1	1	1	8/9
Gao et al., 2024 [37]	1	1	1	-	1	1	1	1	7/9
Dwyer et al., 2024 [38]	1	1	1	-	2	1	1	1	8/9
Alaskarov et al., 2024 [39]	1	1	1	-	2	1	1	1	8/9
Sheikhany et al., 2022 [40]	1	1	0	-	2	0	1	1	6/9
Au et al., 2013 [41]	1	1	0	-	1	0	1	1	5/9
Pei et al., 2019 [42]	1	0	0	-	1	0	1	1	4/9

**Figure 5:**
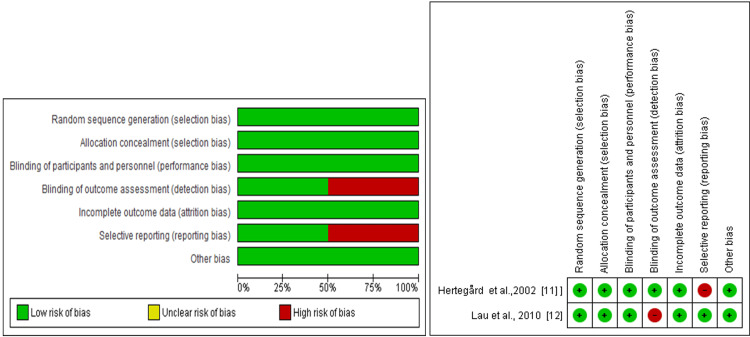
Summary of risk of bias for the items of RCT studies.

For randomized controlled trials (RCTs), the Cochrane Risk of Bias Assessment Tool was applied (Higgins et al. [43]). Figure 5 shows that the two RCT studies assessed for risk of bias using the Cochrane risk of bias assessment tool. Each of the studies had a high risk of bias in one of the seven domains. A study by Hertegard et al. had a high risk of bias in selective reporting, while all other domains had a low risk of bias [11]. On the other hand, a study by Lau et al. had a high risk of bias in blinding the outcome, while other domains of the study had a low risk of bias [12]. By awarding one score in each domain, each of the included studies scored 6 out of the 7 total possible scores in the seven domains. Both studies combined scored 50%-100% in each of the seven domains evaluated. This implies that generally, both studies were of good quality with a low risk of bias in all domains. Therefore, the results from these studies can be reported with confidence.

**Figure 6:**
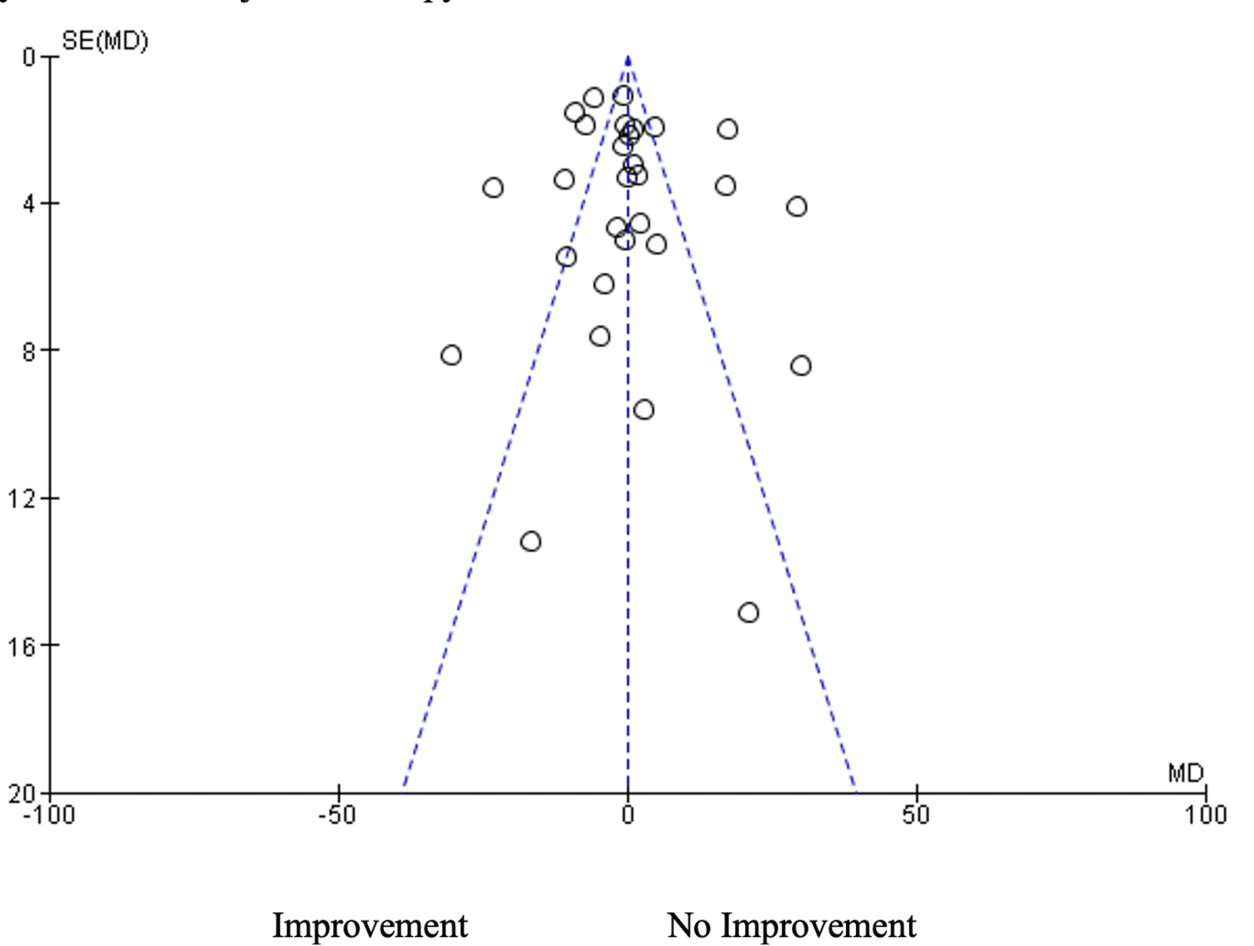
Risk of the publication bias assessment. The funnel plot illustrates asymmetry, suggesting potential publication bias. The included studies are represented by reference numbers [11-42]. SE: Standard Error, MD: Means Difference

The funnel plot in Figure 6 illustrates an asymmetrical funnel where most of the studies are centered at the funnel's center. On the other hand, the left side of the funnel has more outliers than the right. This indicates that there may be a publishing bias that favors patient improvement following hyaluronic acid injection therapy.

Discussion 

UVFP is a neurological condition characterized by glottal insufficiency, often resulting from iatrogenic damage sustained during head and neck surgery [3]. This condition can significantly impact voice quality, swallowing, and overall quality of life [4]. Hyaluronic acid (HA), a naturally occurring glycosaminoglycan, has demonstrated positive effects on vocal cord function by improving glottal closure and restoring vibratory properties [11]. This systematic review and meta-analysis sought to evaluate the use of HA in the treatment of UVFP. The findings demonstrated a significant improvement in patients' overall quality of life, with an odds ratio of 5.73 (95% CI [4.28-9.43]), highlighting the transformative potential of HA injections in managing UVFP (p < 0.00001). This improvement shows the role of HA in addressing not only the physiological but also the psychosocial aspects of UVFP by enabling better vocal function and reducing associated discomfort and distress. Supporting these findings, a study by Hertegård et al. reported substantial enhancements in patient self-ratings and glottal closure 12 months after collagen and Hylan B gel injections [11]. Additionally, Lau et al. revealed that trans cartilage IL with larger particle sizes is safe and durable and may be considered for UVFP patients seeking medium-term intervention [12]. Moreover, a study by Friedman et al. reported that early injection medialization prevented open neck phonological repair in 62.5% of the patients who received the treatment [13].

Multiple studies reported substantial QOL improvements following HA injection. The pooled analysis showed significant benefit with minimal heterogeneity (I²=0%). This consistency across diverse settings suggests that HA laryngoplasty can positively impact subjective outcomes, particularly within the first six months post-treatment. However, long-term QOL outcomes beyond 12 months were reported in only a few studies and remain inconclusive. According to a study by Rudolf et al., 58% of patients who received HL treatment were found to have acceptable voice quality after a year. However, the study noted that after six weeks, 42% of them experienced significant voice impairment requiring further treatment, such as thyroplasty or laryngectomy [14]. Further, Wang et al. found a significant decrease in NGGA, MPT, Voice Handicap Index (VHI), and other evaluated factors, such as the phonation quotient and mean airflow rate scale. Conversely, choking severity was observed after all patients successfully underwent the surgery without any issues [15]. A study by Szkiłkowska et al. found all evaluated cases achieved glottic closure, and after three months, vocal fold vibration reappeared in eight more cases, revealing significant changes in voice quality [16]. Oguz et al. also found a postoperative drop in fundamental frequency, jitter, and noise-to-harmonics ratio. However, no significant differences were observed in the acoustic analysis findings or GRBAS scale parameters [17].

MPT, a critical objective measure of vocal efficiency, improved moderately in the medium-term group. The meta-analysis indicated a statistically significant gain (SMD=0.45), and individual studies supported this trend. Supporting these findings, Upton et al., Halderman et al., Fang et al., and Pei et al. revealed significant improvements in all outcome measures at one and four months. However, research by Upton et al. found that one patient required intravenous steroid therapy for delayed glottic inflammation [18-21].

Improvements in NGGA were also consistent across the included literature. Pooled data indicated a medium effect size (SMD=0.51) with minimal heterogeneity. While imaging-based assessments were not uniformly applied, most studies used stroboscopy or video laryngoscopy to quantify NGGA, enhancing comparability. According to a study by Wang et al., significant changes in glottal closure characteristics were observed after one, six, and the most recent evaluations. These changes included a decrease in the mean normalized gap area, reduced phonation quotient, extended maximum phonation time, and decreased airflow rate [22]. Moreover, studies by Fang et al., Wong et al., Kara et al., and Kim et al. found that following IL therapy with hyaluronic acid, there were notable improvements in self-questionnaire, auditory, visual, and acoustic assessments, in addition to good vocal fold function improvement among patients with UVFP [23-26].

The findings of this study further revealed a significant improvement in the total normalized glottal gap area (NGGA) among participants (p=0.0005). This aligns with results obtained in studies by Ng et al., Pei et al., Bertroche et al., and Liu et al., which noted that HIL effectively treats UVFP. Additionally, the studies reported that the treatment enhances QOL, tone production, pitch characteristics, and perceptual voice quality [27-30]. Studies by Enver et al., Jeong et al., Chow et al., and Chang et al. reported that early transthyrohyoid injection laryngoplasty significantly improved voice and quality of life without causing any major complications in patients with UVFP. Additionally, the treatment was found to be safe and effective. Slightly different from other studies, a study by Enver et al. reported a few complications, predominantly inflammatory reactions with dysphonia and dyspnea, among patients receiving hyaluronic acid under general anesthesia [31-34].

In a study by Miaskiewicz et al., a notable improvement in the glottal gap was reported in both groups, with the CaHA group's mean change in VHI being 29.14 and the HA group's mean change being 22.88 [35]. Moreover, Dwyer et al. found that after three months, Silk-HA continued to show improvement in glottic insufficiency, with overall voice outcomes improving [36]. Nonetheless, studies by Gao et al. and Dwyer et al. demonstrated improvement in glottal closure 12 months following Silk-HA injection. The studies noted that Silk-HA is both a safe and efficient injectable for treating UVFP and augmenting VFI. However, the studies recommended future research to focus on its long-term effects [37-38].

A study by Alaskarov et al. also revealed that among patients with UVFP, HA/D injection laryngoplasty significantly improved voice and swallowing functions (excluding maximal phonation length) in both the short and medium term. However, the study noted no significant change between the preoperative value and the postoperative value after a 24-month period [39]. Studies by Sheikhany et al., Au et al., and Pei et al. showed considerable improvement in glottal conformation, voice, and aerodynamics among individuals with UVFP in the short and medium term (one year or less) following hyaluronic acid injection (HIL) [39-42]. This implies that HIL is both safe and effective for short- and medium-term treatment.

Among the shortcomings noted in the study are the lack of reliable information on the potential long-term benefits of the treatment, potential publication bias, and limited data on pediatric patients. Additionally, broad research should be conducted across diverse healthcare settings and patient-specific characteristics such as age, gender, etiology of vocal fold paralysis, and injection technique to increase the generalizability of the findings. Moreover, investigating patient-specific factors that affect recovery will be crucial to improving outcomes and tailoring treatment strategies to individual needs. Finally, while short- and medium-term outcomes demonstrate improvements in quality of life, phonation time, and glottal closure, only a small number of included studies reported outcomes beyond 12 months. This restricts the ability to evaluate the sustained efficacy and durability of HA injection laryngoplasty over time. Therefore, future research should prioritize long-term follow-up studies to better understand the persistence of treatment benefits and to guide long-term clinical decision-making.

## Conclusions

In conclusion, this systematic meta-analysis found that hyaluronic acid injection significantly improves voice quality and overall quality of life in people with unilateral vocal fold paralysis. Specifically, it enhances glottal closure, maximal phonation time, and patient-reported outcomes. The treatment appears to have short- to medium-term benefits, despite certain disadvantages, including a lack of long-term evidence and patient response variability. Future research should focus on determining long-term efficacy, examining patient-specific characteristics that impact recovery, and exploring previously unreported outcomes. Expanding studies to include more diverse demographics will help individuals with UVFP benefit from customized treatment programs.
